# Impact of a Turbulent Ocean Surface on Laser Beam Propagation

**DOI:** 10.3390/s22197676

**Published:** 2022-10-10

**Authors:** Omar Alharbi, Tim Kane, Diane Henderson

**Affiliations:** 1 Department of Electrical Engineering, The Pennsylvania State University, University Park, State College, PA 16802, USA or; 2 Department of Electrical Engineering, College of Engineering, Majmaah University, Majmaah 11952, Saudi Arabia; 3 Department of Mathematics, The Pennsylvania State University, University Park, State College, PA 16802, USA

**Keywords:** air–sea interface, optical propagation, underwater FSO link, surface waves, refraction, laser beam

## Abstract

The roughness of the ocean surface significantly impacts air-to-sea imaging, oceanographic monitoring, and optical communication. Most current and previous methods for addressing this roughness and its impact on optical propagation are either entirely statistical or theoretical, or are ‘mixed methods’ based on a combination of statistical models and parametric-based physical models. In this paper, we performed experiments in a 50-foot-wave tank on wind-generated waves, in which we varied the wind speed to measure how the surface waves affect the laser beam propagation and develop a geometrical optical model to measure and analyze the refraction angle and slope angle of the laser beam under various environmental conditions. The study results show that the laser beam deviations/distortions and laser beam footprint size are strongly related to wind speed and laser beam incidence angle.

## 1. Introduction

The ocean covers more than 70 percent of the world’s surface. Over the years, people have explored the oceans to satisfy basic scientific curiosity and more pragmatic concerns such as shipping routes and schedules, oil field maintenance, or tactical surveillance. Nevertheless, according to The National Oceanic and Atmospheric Administration (NOAA), about 95% of the oceans remain unexplored [1,2]. To continue monitoring the ocean and marine activities, autonomous underwater vehicles (AUV), remotely operated underwater vehicles (ROV), and other sensors have been deployed to gather, collect, and transmit data about this environment.

The demand for reliable, high-speed communication is accelerating; it is estimated [3] that by 2030 there will be more than 40 billion devices connected to the internet simultaneously. These devices include computers, smart devices, lidars, and, eventually, autonomous underwater vehicles. The concept of the Internet of Things (IoT) was invented in 1985 [4]; in 2012, the Internet of Underwater Things (IoUT) was first discussed. The IoUT is defined as “the network of smart interconnected underwater objects”. The smart objects could be several types of underwater sensors, autonomous underwater vehicles (AUVs), autonomous surface vehicles (ASVs), buoys, ships, etc.

Furthermore, with the rapid development of autonomous underwater vehicles (AUVs) and unmanned aerial vehicles (UAVs), there is an increasing need for reliable communication between these two types of platforms for efficient information exchange. In recent years, considerable work has been directed toward underwater acoustic, RF, and wireless optical technologies. Despite the advances in underwater acoustic communication (UAC), it suffers a significant latency (slow speed of sound in water), which leads to considerable Inter-Symbol Interference (ISI). Thus, UAC links have extremely limited data rates and do not ensure link security for some distances [4,5]. In addition, acoustic signals are affected by the movement of water, like currents or tides. Due to the conductive nature of seawater, RF signals have a higher attenuation in the high frequencies; therefore, most commercial radio equipment (MHz and GHz range) cannot be used underwater. This is the main reason for using very low frequencies (VLF) and extremely low frequencies (ELF). However, the main limitations of these low frequencies are the required large antenna sizes [2].

**Figure 1:**
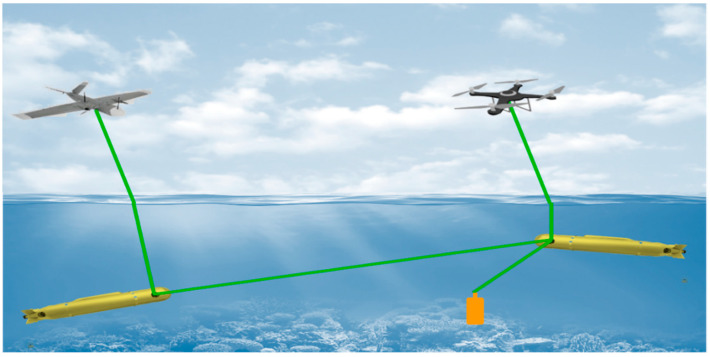
Concept of laser-based communication through both atmospheric and underwater channels.

The limitations of the previous technologies have motivated the development of underwater optical wireless communication (UOWC) with its promise of high data rates over reasonable distances [6]. Optical wireless communication (OWC), also termed free space optics (FSO) [7], is considered an excellent choice for point-to-point communication for the atmospheric channel, the oceanic channel, and the air–sea interface, as shown in Figure 1. The air–sea interface presents an extremely complicated communication scenario because it involves a random path between an atmospheric transmitter and underwater receiver or vice versa, and thus has a considerable impact on not only optical communication but also air-to-sea imaging, laser-line scanning, and other remote sensing systems.

If the air–sea interface were quiescent, then it would be a specular surface with two layers with different refractive indices. However, the air–sea interface is rough due to ocean waves [8], especially wind waves. According to the Beaufort wind scale, wind can be divided into 12 scales or forces [9]. In this paper, our upper limit is Beaufort 3 (wind speed is below 5.5 m/s) because, at this level, the laboratory waves start breaking, which leads to gas bubbles and whitecap generation. As a result, the performance of the optical communication path across the ocean surface will be degraded or even hard to establish [10].

Ocean surface waves are typically measured by two approaches [11]. The traditional method uses in situ buoys, such as those employed by the National Data Buoy Center (NDBC) [12]. A second method is to take instantaneous images of the ocean waves, then process and analyze them to obtain the oceanic wave spectrum. The wave spectrum provides the distribution of wave energy or variance contained over different temporal frequencies and spatial wavelengths on the ocean surface. It can be described by either a one-dimensional (1D) frequency spectrum E(f) or by a two-dimensional (2D) frequency directional spectrum E(f,ϴ) [13]. A review of ocean wave spectra is provided by Ryabkova [14], and a summary of widely used models is given in Section 2.2 below.

In this paper, we present experiments on wind-generated waves, in which we varied the wind speed to measure how the surface waves affect the optical propagation of a laser beam. We considered laser beams with two different diameters from below the glass-bottomed wave tank. Data from specific points on the surface were captured by a high-speed camera above the wave tank. These measurements were compared to established models of ocean wave spectra. The main objective of this work is to understand the interface displacement and slope and assess its subsequent impact on point-to-point FSO communication links above and under the interface.

The rest of the paper is organized as follows. Section 2 briefly reviews ocean wave modeling and some established ocean wave spectra. Section 3 reviews the behavior of the laser beam propagation at the air–sea interface. The experimental apparatus and the methods of data analysis are described in Section 4. Section 5 presents results on the wave spectra and the laser propagation. Section 6 summarizes the conclusions, limitations, and future directions.

## 2. Ocean Wave Considerations

Knowledge of the characteristics of ocean surface waves is required to interpret their measurements. In Section 2.1, we review the description of linear, monochromatic waves at the interface. Section 2.2 reviews established models of ocean wave spectra that use these building blocks in statistical descriptions of the ocean surface.

### 2.1. Monochromatic Capillary–Gravity Waves

A monochromatic, periodic wave on the free surface of water propagating in two horizontal dimensions, *x* and *y* may be described as:

(1) η(x, y, t)=acos(kx+ly−ωt+ϕ)

where *z* = 𝜂 (*x*, *y*, *t*) is the location of the free surface with z=0 corresponding to the quiescent surface and z=−h corresponding to the uniformly horizontal bottom of the fluid column [15]. Here *a* is the wave’s amplitude, k is the *x*-wavenumber, l is the *y*-wavenumber, ω is the frequency, and ϕ is a phase. One can find the relationship between the wavenumbers and frequency from the linearized Stokes boundary value problem [16] for waves at a water surface. Approximating the water as an incompressible, inviscid fluid with irrotational motions, one can describe its velocity vector as the gradient of a scalar potential, φ(*x*, *y*, *z*, *t*). Conservation of mass requires

(2) $$\nabla^{2}\varphi =0\;\;\;\;for\;x\in ℛ,\;y\in ℛ,\;z\in \left(-h,\;\eta \left(x,\;y,\;t\right)\right).\;\;\;\;\;$$

The kinematic boundary condition that defines the free surface is:

(3) $$\frac{\partial \eta }{\partial t}-\frac{\partial \varphi }{\partial z}+\frac{\partial \eta }{\partial x}\;\frac{\partial \varphi }{\partial x}+\frac{\partial \eta }{\partial y}\;\frac{\partial \varphi }{\partial y}=0\;\;\;\;at\;z=\eta \left(x,\;y,\;t\right)\;.\;$$

The dynamic boundary at the free surface is that the curvature balances the pressure jump due to surface tension so that:

(4) $$\frac{\partial \varphi }{\partial t}+g\eta +\frac{1}{2}\left[{\left(\frac{\partial \varphi }{\partial x}\right)}^{2}+{\left(\frac{\partial \varphi }{\partial y}\right)}^{2}+{\left(\frac{\partial \varphi }{\partial x}\right)}^{2}\right]=\frac{T\;\nabla^{2}\eta }{\;{\left(1+{\left(\frac{\partial \eta }{\partial x}\right)}^{2}+{\left(\frac{\partial \eta }{\partial y}\right)}^{2}\right)}^{3/2}}at\;z=\eta \left(x,\;y,\;t\right),\;$$

where 𝑇 is the kinematic surface tension, and *g* is the acceleration of gravity. The boundary condition at the bottom is that there is no flow through the bottom. The vertical velocity vanishes there so that:

(5) $$\frac{\partial \varphi }{\partial z}=0\;\;at\;z=-h\left(x,\;y,\;t\right).$$

The solution of the linearized version of this boundary-value problem, along with (1), is that the velocity potential is:

$$\varphi \left(x,\;y,\;z,\;t\right)=a\frac{\omega }{\kappa }\sin\left(kx+ly-\omega t+\phi \;\right)\frac{\cosh\left[\kappa \left(z+h\right)\right]}{\sinh\left[\kappa h\right]},$$

and the frequency and wavenumbers are related by the dispersion relation,

(6) $$\omega^{2}=g\kappa \left(1+\frac{T\kappa^{2}}{g}\right)\tanh\left(\kappa h\right),$$

where $\kappa =\sqrt{\kappa^{2}+l^{2}}$ is the 2D wavenumber. The corresponding wave phase speed is

(7) $$C_{p}=\omega \left(\kappa \right)/\kappa$$

and, in general, may depend on the wavenumber. Several classifications of water waves are obtained from Equation (6).

1. The first classification is with respect to the depth through the size of κh. For κh≫ 1, tanh(κh)≈1, the effects of depth are neglected, and the result is “deep-water waves’’, also called “short waves’’. For these waves, $C_{p}$ is a function of wavenumber, so waves with different wavelengths travel at different speeds. Thus, deep-water waves are dispersive waves. The waves discussed in this paper are deep-water waves. For κh≪1, tanh(κh) ≈κh, and the results are “shallow-water waves”, also called “long-waves’’. For these waves, $C_{p}$ is approximately independent of wavenumber. Thus, shallow-water waves are approximately non-dispersive. For κh=1, there is no approximation on tanh(κh). Such waves are dispersive.
2. The second classification is with respect to the Bond number [17]:
  (8) $$\beta =\left(\frac{T\kappa^{2}}{g}\right),$$
  which measures the relative importance of capillary forces versus gravitational forces. For β = 1 (using $T=73.0\;\frac{cm^{3}}{s^{2}}$ and $g=981\;\frac{cm}{s^{2}}$), the wavelength is a critical value of 1.71 cm, and the two restoring forces balance. This wavelength corresponds to the minimum phase speed of about $C_{p}$ = 23.1 cm/s for deep-water waves. For β ≫ 1, the wavelengths are shorter than the critical value, and capillary forces dominate. The dispersion relation for these capillary waves is well approximated by:
  (9) $$\omega^{2}=T\kappa^{3}\tanh\left(\kappa h\right).$$
  For β ≪ 1, the wavelengths are longer than the critical value, and gravitational forces dominate. The dispersion relation for these gravity waves is well approximated by:
  (10) $$\omega^{2}=g\kappa \tanh\left(\kappa h\right).$$
3. A third classification takes into account weak nonlinearity. In particular, Equation (6) holds when the wave slope is very small, aκ→ 0. If one allows for finite but weak nonlinearity so that aκ ≪ 1, one finds that capillary–gravity waves and capillary waves may spread energy spectrally through resonant triad and quartet interactions as well as modulational instabilities [18]. Gravity waves on finite depth or deep water spread energy through modulational instabilities and resonant quartet interactions. Thus, even in the absence of wind, these instabilities and interactions may cause complicated two-dimensional surface patterns [19] from freely propagating waves.
4. A fourth classification is with respect to the presence or absence of wind-forcing. Waves are classified as being either “sea” or “swell”, where seas are the waves that feel the influence of wind-forcing, and swelling are the waves that have propagated away from the influence of the wind. Because of the wind-forcing, seas are steeper than swells; they have a larger value of aκ than the swells. Because deep-water waves are dispersive, the swells sorted themselves into narrow-banded spectra, with the longer waves traveling faster than the shorter waves. Their frequencies are smaller, and their wavelengths are longer than those of the sea. A typical separation frequency is about 0.18 Hz [20]. The waves in the experiments discussed herein are seas. Their spectra are broad-banded and propagate in two horizontal directions at the air–sea interface. They have two independent slopes: the along-wind slope formed along the wind direction (*x*-axis herein) and the cross-wind slope formed perpendicular to the wind direction (*y*-axis).

In summary, the waves considered herein are seas comprised of deep-water, capillary–gravity waves of finite amplitude. In addition, they are fetch-limited. Fetch is the distance over which the wind blows. The minimum duration time, $t_{min}$ is the time for waves to travel from the beginning of the fetch to a distance, *r*. If the wind blows for a time larger than $t_{min}$, then the wave height at the position, *r*, stops growing, and the waves there reach (on average) a steady-state height [21]. For the experiments herein, measurements are obtained for times, *t* > $t_{min}$.

### 2.2. Models of Ocean Wave Spectra

Waves on the ocean surface are not monochromatic and are typically modeled as a superposition. The wind puts energy into a broad spectrum of wave modes so that one may write the surface displacement in terms of a Fourier Transform:

(11) $$\eta \left(x,y,t\right)=\int_{-\infty }^{\infty }\int_{-\infty }^{\infty }A\left(k,l,\omega \right)e^{-i\left(kx+ly-\omega t\right)}dk\;dl$$

where k and l are the *x* and *y* wavenumbers, ω is the corresponding frequency, A(k,l,ω) is the Fourier amplitude, and one adds the complex conjugate to obtain real values for the free surface displacement.

Such a representation is too complicated because one needs complete information about the storm that generated the waves to solve for A. In addition, this view does not account for wave damping primarily due to wave breaking and energy transfer due to nonlinear interactions during propagation. Instead, investigators have developed a wide variety of models for spectra [14]. Pierson–Moskowitz [22] used similarity theory to find a closed-form representation of the frequency spectrum for fully-developed seas, a situation in which the energy input by wind and the dissipation due to breaking are balanced. The Pierson–Moskowitz energy spectral density is

(12) $$\;\;S_{PM}\left(\omega \right)=\frac{\alpha g^{2}}{\omega^{5}}e^{\left[-\delta {\left(\frac{\omega_{0}}{\omega }\right)}^{4}\right]}$$

where α = 8.1 × 10^−3^, δ = 0.74, $\omega_{0}=g/U$, and U is the wind speed measured above the surface, typically at 10 m, if possible. This speed, U, is used since the friction velocity at the interface is not measured. However, data obtained during the Joint North Sea Wave Observation Project (JONSWAP) [23], showed that the ocean wave spectrum is typically not fully developed. To account for nonlinear interactions and the fetch-dependent balance of energy sources, sinks, and energy transfer, the authors in [21] modified the Pierson–Moskowitz spectrum and used data from the project to develop the JONSWAP spectrum,

(13a) $$S_{J}\left(\omega \right)=\frac{\alpha_{J}g^{2}}{\omega^{5}}e^{\left[-\frac{5}{4}{\left(\frac{\omega_{p}}{\omega }\right)}^{4}\right]\gamma^{r}}$$

(13b) $$r=e^{\left[-\frac{{\left(\omega -\omega_{p}\right)}^{2}}{2\sigma^{2}\omega_{p}^{2}}\right]}$$

which varies with respect to *U* (measured 10 m above the surface in reference to JONSWAP paper) and *x*, the fetch. Here:

αJ=0.076(U102xg)0.22, ωp=22(g2U10x)13, γ=3.3, σ={ 0.07 ω≤ωp 0.09 ω>ωp

where $\omega_{p}$ is the frequency at the spectrum’s peak, and g is the acceleration of gravity. The inclusion of *x* allows the spectrum to vary with distance from the wind source, as per the data obtained in [24].

A summary of several significant wave models and spectra is listed in Table 1. In Section 5.2, Figure 14, we compare measured spectra obtained from laser measurements to those obtained using the PM and JONSWAP spectra.

## 3. Laser Propagation at the Air–Sea Interface

Numerous researchers have carried out theoretical and experimental investigations of oceanic surface waves’ influence on laser propagation. The relationship between sea surface conditions and the accuracy of airborne LiDAR bathymetry (ALB) was investigated at [10,34,35]. In [1], they experimentally demonstrated high-speed optical wireless communication for both the uplink and downlink by employing an OFDM transmission of 32-QAM and single-mode pigtailed green-light laser diode (LD). Although a data rate of 5.5 Gbps was achieved over a 26 m air–water link with accurate pointing between the transmitter and the receiver, their experiment assumed the oceanic surface was static.

Wang et al. [36] investigated the turbulent propagation of radial partially coherent beams and Gaussian Schell model beams in an air–sea hybrid link scenario while ignoring the turbulent interface. A fast analysis method was proposed to compute the transmittance and the refraction angles through a wavey interface in different wind directions and speeds by simulating the wind-driven dynamic waves for lidar application [37]. The impact of water height rather than wavy interface was investigated at [38]. In [39], they demonstrated a diffuse-line-of-sight communication link through a wavy interface by UV LED as a signal carrier. Majmudar [30] proposed a probabilistic model for the refraction angle of optical propagation at the random air–water interface. A high-speed system for direct optical communications across a water–air interface in a real environment was designed and tested in [40]. AmphiLight was presented and proposed in [41] to enable bidirectional link air–sea interface. Adib [42] proposed hybrid acoustic-RF wireless communication through the water–air interface. For simplicity, the triangular wave facet model was presented and simulated in [43,44,45] using MC ray tracing to compute the sea surface optical reflectance and transmittance after intersecting the interface.

Zhang [46] investigated the effect of large sea surface scale facets on EM scattering by using a capillary wave modification facet scattering model. However, to our knowledge, theoretical and experimental investigations for characterizing the micro-oceanic facets and their influence on laser beam propagation through the interface have not been reported previously.

**Figure 2:**
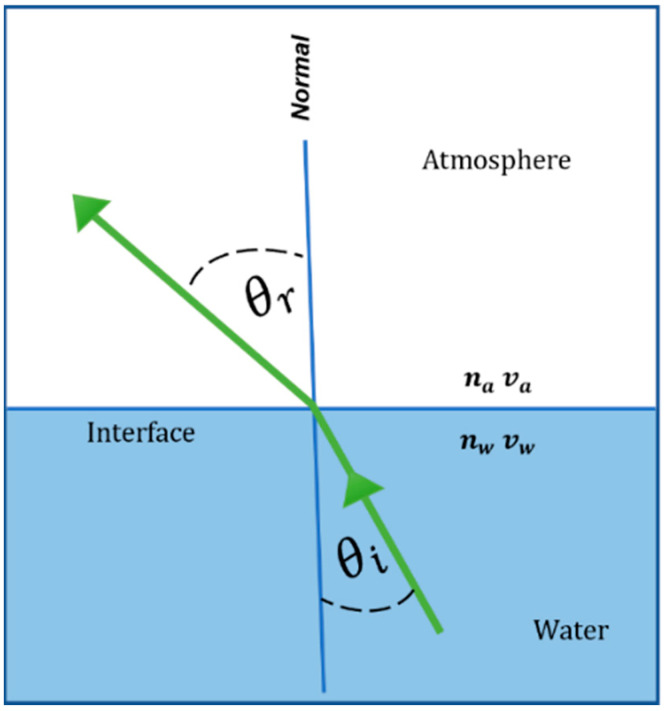
Snell’s Law. $n_{a}\;$and $n_{w}\;$ represent the index of refraction of the air and water channels, respectively. $v_{a}$ and $v_{w}$ represent the velocity of light through air and water, respectively.

When a laser beam propagates from underwater to the atmosphere will change direction and likely beam shape due to refraction, as illustrated in Figure 2. The Law of Refraction (Snell’s Law) explains the relationship between the incident angle and the refraction angle when the light passes through different media, such that,

(14) $$\frac{\sin\theta_{r}}{\sin\theta_{i}}=\frac{n_{a}}{n_{w}}=\frac{\;v_{a}}{v_{w}}$$

Irregular (random) ocean surface waves, which are generated by winds, are the major factor in changing beam direction beyond the smooth surface deviation seen in Equation (14) after passing the air–sea interface [47].

## 4. Experimental Apparatus and Procedures

**Figure 3:**
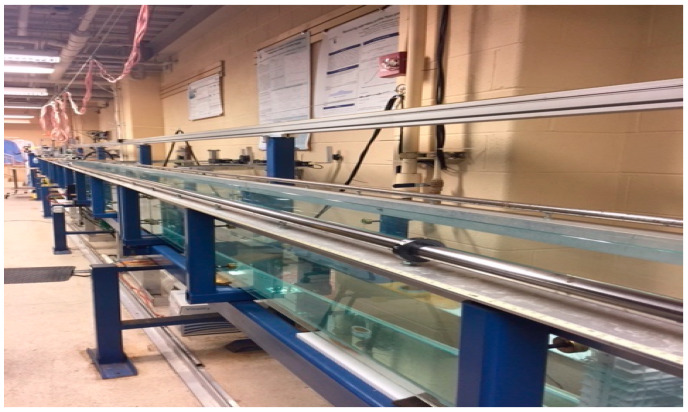
William G. Pritchard Fluid Mechanics Laboratory at the Mathematics Department at Pennsylvania State University.

The experimental apparatus comprised a wave tank, a fan assembly, a laser assembly, and a photographic assembly. Figure 3 shows a photograph of the wave tank. It is 50 ft long, 10 in wide, and can be filled up to 12 in.

We filled it with tap water to a depth of 20 cm for these experiments. The tank has precisely aligned glass sidewalls and a bottom supported by a steel structure. The wind tunnel fits into the wave tank, with its vertical position adjustable to be the desired height (about 1 cm) above the water surface. It used a Can Max Fan Mixed Flow Inline Fan with a 10-inch duct diameter that blows up to 1023 ft^3^/min and rotates at a speed up to 2990 rpm. The fan blows into an enclosed Plexiglas chamber the same width as the tank and is 25 cm high and 25 cm long. The downstream wall is a gate with an array of holes. The roof has a slit with a door. We closed the gate and opened the slit so that when the wind was turned on, it blew the air up toward the ceiling, not over the water surface. The fan ramped up to the desired wind speed over about 10 s. After it reached its steady state, we closed the slit and opened the gate simultaneously to create an impulsive start for the wind over the water surface. The air blew from the chamber through three layers of filter material, through a honeycomb of tubes 2.5 in long, then over the water. The airspeed was measured using a TSI, 8465-12 anemometer, and the wind speed value, U, was obtained at a desired height above the water surface for all experiments. While the goal was to investigate how a wavy air–sea interface affects laser propagation, the experiments also provided data on wind-generated waves.

**Figure 4:**
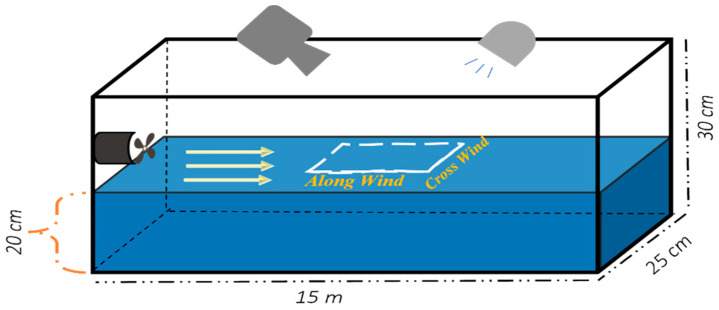
Schematic layout for modeling the air–water interface where the diffused light source is placed in the opposite direction for the water flow and the high-speed camera with the same wind direction.

To obtain visual data on wind-generated waves, a light source and a Photron FASTCAM Mini UX UX100 high-speed camera were placed above the wave tank at a specific angle in the “front” and “back” of the wave direction, respectively. Figure 4 shows a schematic of the setup for these experiments. We used three wind speeds (maximum at 3.3 m/s, medium at 1.9 m/s, and minimum at 0.9 m/s). We took instantaneous images as a video stream of a portion of the air–sea interface, shown as the dashed square in Figure 4, for five to seven seconds at speeds of 1 kf/s and 4 kf/s. The images were compared to those of the flat interface (no wind) to determine the displacement of the air–sea interface.

**Figure 5:**
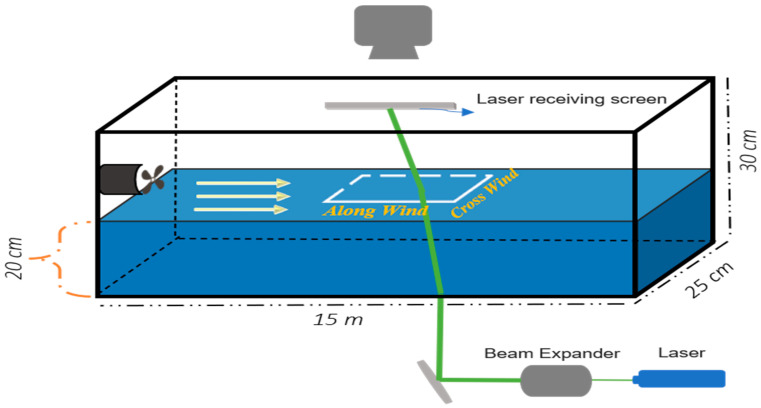
Schematic layout for investigation of laser propagation through the interface.

A doubled Nd:YAG laser operating at 532 nm wavelength was placed below the wave tank with a beam expander to study the effects of laser propagation through a wavy interface. After the expansion process, the beam was reflected by a mirror to obtain the desired location and angle at the receiver. A high-speed digital camera was located at the top of the wave tank to capture the laser beam footprint on a translucent screen 27 cm above the air–water interface. Figure 5 shows a schematic of the set-up for these experiments.

**Figure 6:**
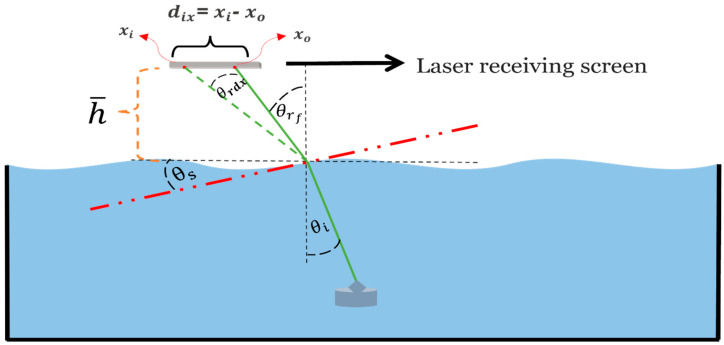
Schematic diagram of the light path through a rough ocean surface. $x_{o}$,$x_{i}$ represent the laser beam centroid at the quiescent interface and at frame i, respectively. The laser receiving screen is placed at an approximated mean height $\bar{h}$ = 27 cm. $\theta_{i}$ and $\theta_{r_{f}}$ are the incident and refraction angles at the quiescent interface case. The deviation in the refraction angle in the along-wind direction $\theta_{r_{dx}}$ depends on the slope angle $\theta_{s}$ averaged over the beam footprint at the turbulent interface.

To analyze the laser propagation through the random interface, a geometric optical model is considered and shown in Figure 6. Let $\theta_{i}$ be the incident angle underwater, $\theta_{rf}$ be the refraction angle at a quiescent interface, $\theta_{r_{d}}$ be the deviated refraction angle at time $\left(t_{i}\right)$. The deviations of these angles in the along-wind and cross-wind (assuming independence) directions can be computed using right triangles as follows. The right triangle in the *xz*-plane with perpendicular sides $di_{X}$ and $\bar{h}$ give the angle in the along-wind direction as:

(15) $$\theta_{rdx}={tan}^{-1}\left(\frac{di_{X}}{\bar{h}}\right)$$

Similarly, the right triangle in the *yz*-plane (out of the page) with perpendicular sides $di_{Y}$ and $\bar{h}$ give the angle in the cross-wind direction as:

(16) $$\theta_{rdy}={tan}^{-1}\left(\frac{di_{Y}}{\bar{h}}\right)$$

where $\theta_{rdx}$ and $\theta_{rdy}$ represent the change in the refraction angle from that of the quiescent interface in the along-wind and cross-wind directions, respectively.

The terms $di_{X}$ and $di_{Y}$ are the differences between the laser beam centroid “center of mass” at frame i and the centroid for the reference frame along the *x*-axis (along-wind direction) and *y*-axis (cross-wind direction), respectively. The $\bar{h}$ value represents the height of the target screen. Then, the total refraction angle in the along-wind direction can be written as:

(17) $$\theta_{r_{aw}}=\theta_{rf}\pm \theta_{rd_{X}}$$

Similarly, for the cross-wind direction, the total refraction angle is:

(18) $$\theta_{r_{cw}}=\theta_{rf}\pm \theta_{rd_{Y}}$$

Snell’s law Equation (14) must be modified to include the interfacial slope $\theta_{s}$ and so one may determine its angle, as shown in Figure 6. We can then rewrite Equation (14) in the following form:

(19) $$n_{w}sin(\theta_{i}+\theta_{s})=n_{a}sin(\theta_{r}+\theta_{s})$$

Using Equation (19), the slope angle is calculated as:

(20) $$\theta_{s}={tan}^{-1}\left(\frac{\left(n_{w}sin\theta_{i}-n_{a}sin\theta_{r}\right)}{\left(n_{a}cos\theta_{r}-n_{w}cos\theta_{i}\right)}\right)\;$$

## 5. Results and Discussion

Here we present data on the two sets of experiments. For the first, we measured the interface displacement from the high-speed camera above the water in front of the wave direction and the diffuse light source in the back of the wave direction (and vice versa) versus three different wind speeds. In the second, we used a laser beam with an expander placed below the wave tank at two different incident angles at the air–water interface (normal incidence $\theta_{i}=0^{{}^{\circ}}$ and off-normal incidence $\theta_{i}=32^{{}^{\circ}}$) and the camera placed above the tank, facing $90^{{}^{\circ}}\;$ downward to capture the laser footprint projected on the receiving screen placed above the surface.

### 5.1. Water Surface Spectrum

**Figure 7:**
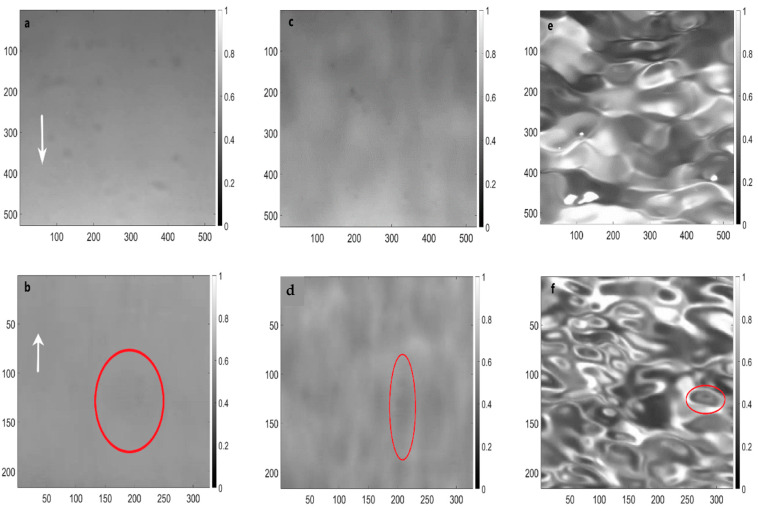
Samples of captured images of the interface at different wind speeds: 0.9 m/s wind speed (**a**,**b**), 1.9 m/s wind speed (**c**,**d**), and 3.3 m/s wind speed (**e**,**f**). The white arrows indicate the wind direction, and the red ovals represent examples of facet sizes at different wind speeds. The incident angle of the diffused light source was $45^{{}^{\circ}}$ for the upper row and $0^{{}^{\circ}}$ for the bottom row.

Since oceanic waves and our laboratory waves have random/stochastic, but not necessarily isotropic motions, the statistical measurements of the air–sea interface are different under differing experimental configurations. Therefore, the illumination of the water facets depends on the incident angle, $\theta_{i}$, of the diffused light source and the refraction from the interface into the camera to obtain the wave spectrum [25]. Thus, locating the high-speed camera above the wave tank with wave direction (forward) produces different spectra from when the camera is located in the opposite direction (backward). Figure 7 shows snapshots of the air–sea interface for both cases at the three wind speeds.

**Figure 8:**
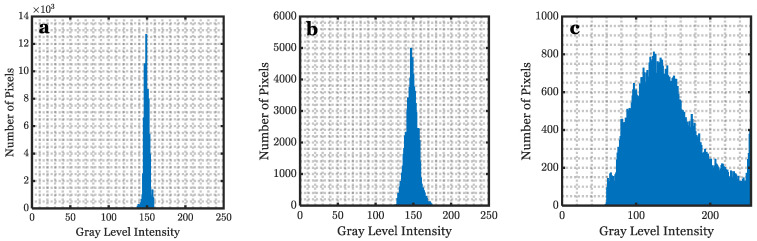
Histograms of pixel intensity for 216 × 318 gray scale images at different wind speeds 0.9 m/s (**a**), 1.9 m/s (**b**), and 3.3 m/s (**c**).

Increasing wind speed increases the surface roughness scale, which creates more oceanic surface elements (facets). Each of these individual facets (depending on their number and size) has a slope and tilting angle that is important to understand when the laser propagates and refracts through the air–sea interface [9,48]. In Figure 7a,b, the white arrow indicates the wind direction. In Figure 7b,d,f, the dashed lines represent example facet sizes at a minimum, medium, and maximum wind speed, respectively. The average facet size was measured to be approximately 8.3 cm^2^, 4.9 cm^2^, and 2.1 cm^2^ for minimum, medium, and maximum wind speeds, respectively. Figure 8 compares the intensity distribution histogram of the pixel intensity values for the backward case at the three wind speeds that were shown in Figure 7b,d,f. From the histogram, the abscissa presents the grey level intensity, which graphically displays 256 numbers showing the distribution of pixels amongst those greyscale values where 0 means the image is purely black, and 255 means the image is overexposed. The ordinate shows how many pixels there are in each case.

Figure 8a, where U = 0.9 m/s, shows that there are very few numbers of facets distributed in a very tight range of gray level intensity (focused from 140 to 165), consistent with Figure 7a,b. As the wind speed increases, the facet size decreases, and their number increases. For instance, in Figure 8c where U = 3.3 m/s, the facet size decreases rapidly while the facet quantity increases, which results in a large variant in the number of facets (with different slopes). To sum up, the shape of the histogram depends on $\theta_{s}$ of each facet and $\theta_{i}$. It should be noted that most of the bright spots are due to the reflection of the diffused light source at random slopes in Figure 7f.

### 5.2. Laser Propagation

**Figure 9:**
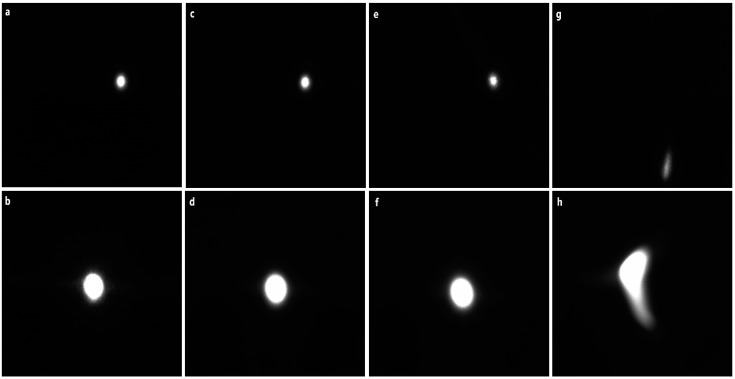
Random samples of laser spots at $\theta_{i}=0^{{}^{\circ}}$ with no wind (**a**,**b**), 0.9 m/s wind speed (**c**,**d**), 1.9 m/s wind speed (**e**,**f**), and 3.3 m/s wind speed (**g**,**h**). The top row is the original beam with a diameter of 0.3 cm, and the bottom row is the expanded beam with a diameter of 0.8 cm.

During the experiments, a high-speed camera is located on the top of the wave-tank just behind the translucent receiver plate (whose height is 27 cm) to capture instantaneous images of the laser beam footprint after passing the air–water interface. The parameters considered in this study are incident angle $\theta_{i}$, wind speed U, and laser beam diameter d. For each configuration, 8700 images were captured and processed to calculate the beam centroid and compare it with a free turbulent interface. The centroid coordinates were calculated based on an image moment algorithm [10,49,50]. The displacement changes in the x-direction (along-wind) or y-direction (cross-wind) from the centroid coordinate of the flat surface show how the laser beam refracted at the surface. To quantify the refraction, we converted each coordinate to a deviation angle from the flat surface’s refraction angle, as shown in Figure 6. To better investigate the influence of the ocean facets on the laser propagation at different wind speeds, a collimated 0.3 cm beam was used as the input beam into the beam expander system to obtain an expanded beam of 0.8 cm. Samples of the original beam and the expanded beam are displayed in Figure 9.

**Figure 10:**
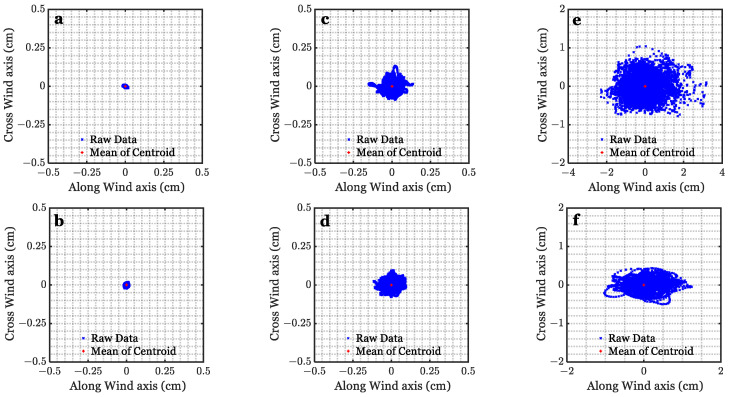
Centroid distribution of the laser spots at $\theta_{i}=0^{{}^{\circ}}$ with 0.3 cm beam diameter (upper row) and 0.8 cm beam diameter (bottom row) at 0.9 m/s wind speed (**a**,**b**), 1.9 m/s wind speed (**c**,**d**), and 3.3 m/s wind speed (**e**,**f**).

The shape of the laser spots in Figure 9c,d for which U = 0.9 m/s are comparable to Figure 9a,b for the quiescent interface. The drift of the laser spot can be observed when the wind speed increases, as shown in Figure 9e,f, for which U = 1.9 m/s, and Figure 9g,h, for which U = 3.3 m/s. Based on the laser beam footprints and their deviation, the variation in the laser beam centroid location was calculated. Figure 10 shows the centroid distribution of the laser beam at $\theta_{i}$ = 0° at different wind speeds for two different values of d.

**Figure 11:**
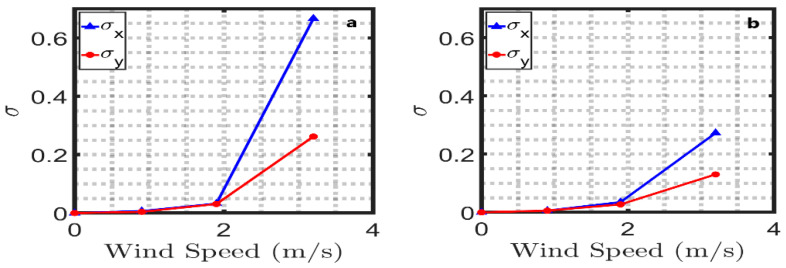
The standard deviation in the along-wind direction (σ_x_) and cross-wind direction (σ_y_) direction with 0.3 cm beam diameter (**a**) and 0.8 cm beam diameter (**b**) at different wind speeds at $\theta_{i}=0^{{}^{\circ}}$.

The centroid location is slightly changed at minimum wind speed in Figure 10a,b and medium wind speed in Figure 10c,d due to the capillary waves on the interface. However, when the wind speed increases, the wavelength increases, and gravity waves are generated. Therefore, the centroid locations drift rapidly in Figure 10e,f compared to those resulting from the minimum and medium wind speeds. Moreover, the deviation in the along-wind direction is higher than in the cross-wind. Figure 11 presents the standard deviation of the centroid drift at different wind speeds at $\theta_{i}=0^{{}^{\circ}}$. The standard deviation is almost equal for a beam diameter of 0.3 cm in Figure 11a and a beam diameter of 0.8 cm in Figure 11b at the minimum and medium wind speeds. On the contrary, since the beam size in Figure 9a is smaller than the facet size at the higher winds, as pointed out in Section 2.1 in Figure 7f, the standard deviation for the along-wind and the cross-wind for a beam diameter of 0.3 cm in Figure 11a is double that exhibited by the 0.8 cm beam diameter in Figure 11b.

**Figure 12:**
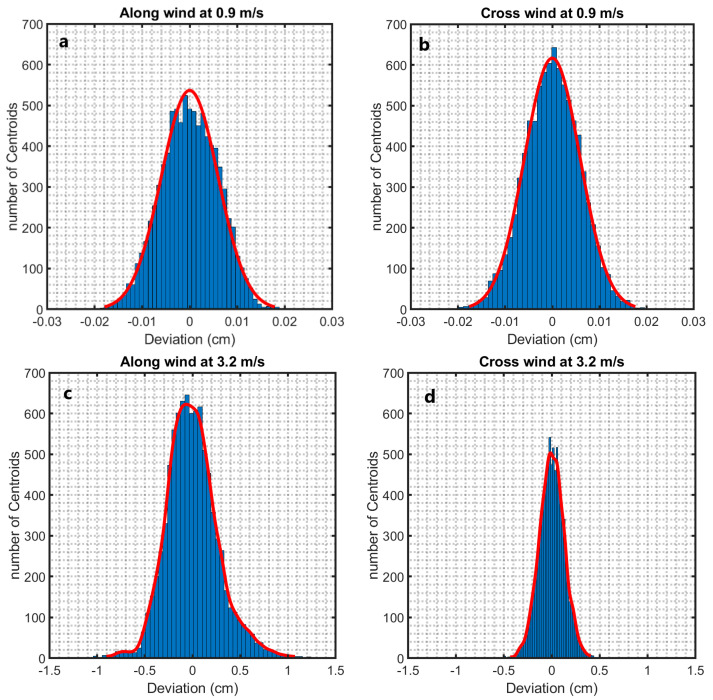
Histogram of centroid distribution of laser beam with *d* = 0.3 cm at $\theta_{i}=0^{{}^{\circ}}$ with 120 cm fetch at (**a**) along-wind direction at U = 0.9 m/s, (**b**) cross-wind direction at U = 0.9 m/s, (**c**) along-wind direction at U = 3.3 m/s, and (**d**) cross-wind direction at U = 3.3 m/s.

Another way of looking at the along-wind and cross-wind displacements of the laser beam centroid due to waves is shown in Figure 12. The histograms there show, on the ordinate, the number of images (that is, the number of centroids) in which the laser beam centroid had a deviation of a value given on the abscissa. Due to the direction of the water flow, which moves parallel to the wind direction as described in Figure 5, the deviation of the displacement centroid from the quiescent interface along the wind direction outpaces the deviation in the cross-wind axis. Figure 12c,d show that the fluctuation increases as the wind speed increases. For U < 1.9 m/s, the histograms in the along-wind and the cross-wind directions nearly follow a Gaussian distribution as shown in Figure 12a,b.

Contrastingly, the deviation of the displacement centroid for maximum wind speed (U < 1.9 m/s) does not fit a Gaussian distribution. Instead, a Gaussian Kernel-smoothing distribution [51] provides the best fit, as shown in Figure 12c,d. Furthermore, the laser beam diameter impacts the deviation of the displacement centroid according to the facet size, as discussed earlier. For instance, the beam centroid with a diameter of 0.8 cm deviates from the still surface case by ±0.02 cm for U = 0.9 m/s in both the along-wind and the cross-wind directions. The deviation for maximum speed is measured as ±1 cm and ±0.5 cm in the along-wind and the cross-wind directions, respectively. For the laser beam diameter of 0.3 cm, the deviation increases. The fluctuation of the centroid measured in the along-wind direction is ±3 cm.

When the laser beam passes the air–sea interface through a single facet, its new direction and path are subject to the along-wind and cross-wind slopes belonging to that facet. The deviation of the refraction angles $\theta_{r}$ and slope angles $\theta_{s}$ in the along-wind axis and the cross-wind axis are calculated and obtained by a centroid shift between each sample and the original quiescent interface, as discussed in Section 2. The standard deviation of the slope angles and the refraction angles for two different beam diameters (0.3 cm and 0.8 cm) and two different angles of incidence ($0^{{}^{\circ}}$ and $32^{{}^{\circ}}$) at different wind speeds are shown in Table 2 and Table 3. These statistical results are in good agreement with those of a previous report [9,10] in which the deviation of both the refraction angles $\theta_{r}$ and slope angles $\theta_{s}$ in the along-wind direction was found to be higher than in the cross-wind direction.

**Figure 13:**
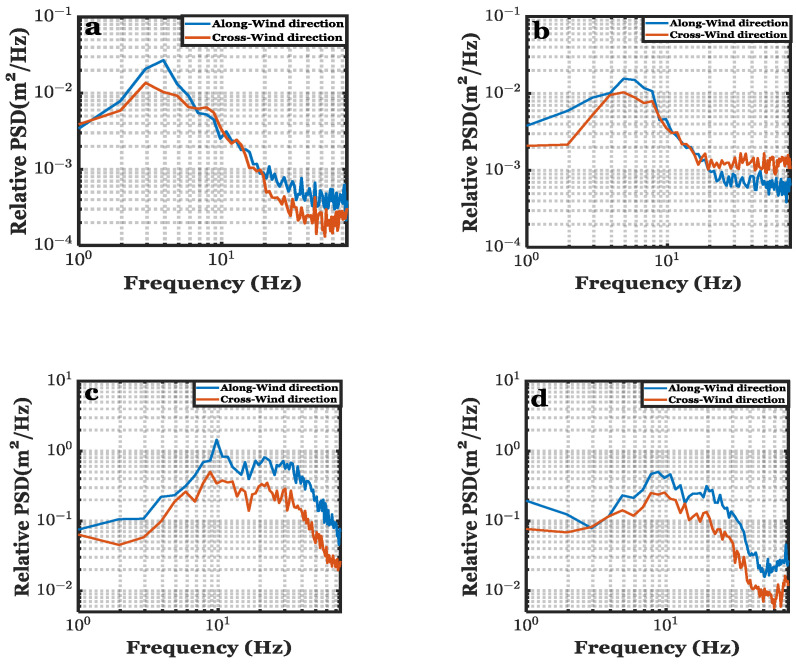
One-dimensional temporal spectrum of beam deviation of *d* = 0.3 cm at U = 0.9 m/s wind speed (**a**), *d* = 0.8 cm at U = 0.9 m/s (**b**), *d* = 0.3 cm at U = 3.3 m/s (**c**), *d* = 0.8 cm at U = 3.3 m/s (**d**). The measurement duration for the experiment presented in this figure was about 8 s.

Based on the centroid measurements that were discussed in the previous section, the temporal power spectral density (PSD) was obtained in order to further analyze he motion of the air–sea interface. Each realization consists of 8734 time samples (frames), and these samples were divided into eight subsets, each consisting of 1024 samples. By using the fft function in MATLAB, the average of these PSDs was then computed. Temporal spectra (both along-wind and cross-wind) of the interface using the lowest and highest wind speeds and the beam diameters of 0.3 cm and 0.8 cm are shown in Figure 13.

Figure 13 shows that the relative power density of the along-wind components is always higher than that of the cross-wind components, especially at lower frequencies. Further, the laser beam diameter does not change the PSDs calculations qualitatively. For example, Figure 13a,b represent the PSD at minimum wind speed for beam diameter *d* = 0.3 cm and *d* = 0.8 cm, respectively. Their peak energy is located approximately at the same frequency, which is *f*~4 Hz. Similarly, Figure 13c,d represent the PSD at maximum wind speed (3.3 m/s) for beam diameter *d* = 0.3 cm and *d* = 0.8 cm, respectively. Their peak energy is located at approximately the same frequency, which is around *f*~10 Hz.

**Figure 14:**
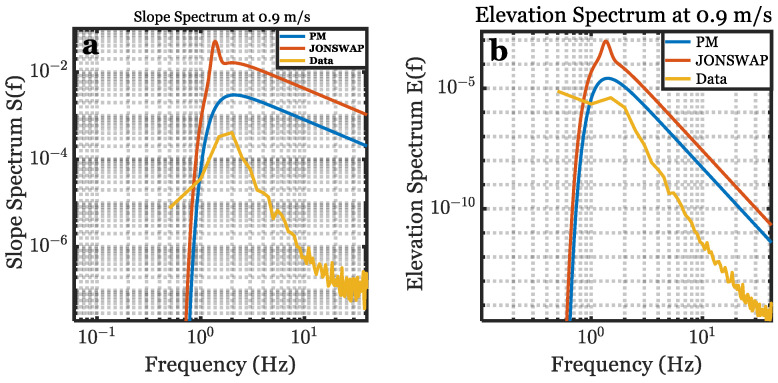

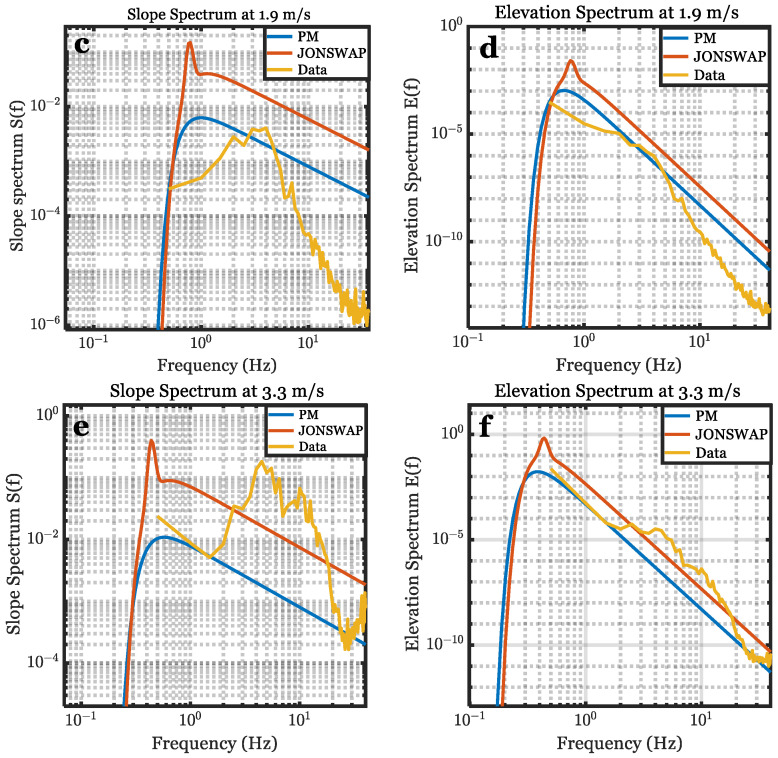
The slope spectrum (left column) and elevation spectrum (right column) for the empirical data, PM, and Jonswap for a fetch of x = 1.2 m at U = 0.9 m/s (**a**,**b**), U = 1.9 m/s (**c**,**d**), and U = 3.3 m/s (**e**,**f**). The measurement duration for the experiment presented in this figure was about 8 s.

**Figure 15:**
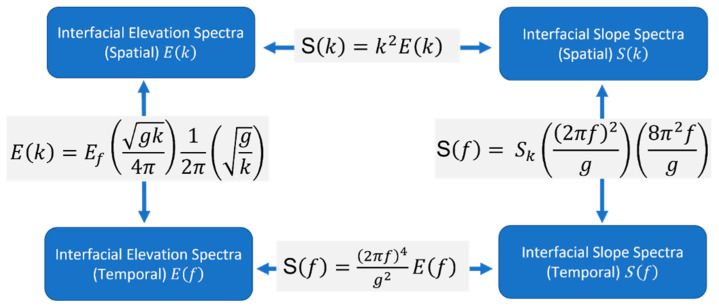
Temporal and spatial frequency conversion for both the slope and elevation spectra.

The spectra shown in Figure 14 are for the interfacial slopes and approximated interfacial elevation. The equations in Figure 15 (see [9,44,52,53,54,55]) incorporate a linear approximation to obtain the approximated 1-D elevation wave spectrum E(f).

The measurement duration for each experiment presented in Figure 5 was about 8 s (8700 frames of data). The interfacial slope spectrum and the corresponding interfacial elevation spectrum for our data were compared to the PM and JONSWAP spectra presented in Table 1 by applying similar parameters in Figure 5. At the lower wind speed, the peak frequency for the slope and the elevation spectra for our data are close to both models, as in Figure 14a,b, respectively, owing to the spectral peak enhancement factor for JONSWAP to improve the spectrum accuracy at the lower frequency [24] The peak frequency for our data tends to increase as the wind speed increases. Contrarily, the peak frequency of PM’s model and JONSWAP’s model decrease as the wind speed increases. The temporal slope spectrum can be converted approximately from the temporal elevation spectrum by $\frac{{\left(2\pi f\right)}^{4}}{g^{2}}$. Hence, the slope spectrum falls off slower than the elevation spectrum at the higher frequencies. The temporal and spatial frequency conversions for both the slope and elevation spectra are shown in Figure 15.

## 6. Conclusions

In this work, we conducted a set of experiments to study dynamic oceanic waves and their influence on laser propagation. We investigated the statistical behavior of irregular small-scale, air–sea interface facets under different configurations. The results show that the facet area decreases when the wind speed increases, which results in raising the slope angle of each facet. In addition, a water–air optical system was built to investigate the propagation of laser beams impacted by the turbulent interface. The results indicated that the offset deviation in the along-wind direction escalates more than in the cross-wind direction with the wind speed. As a result, the standard deviations of the refraction angle, as well as the slope angle, increase.

One limitation of this study that will be addressed in future work was the maximum wind speed of only 3.3 m/s. Improving this model by investigating the laser propagation with bigger beam diameters (larger than the facet size) at the interface with higher wind speeds will enable reliable models which apply to the corresponding larger dynamic range, especially at very lower frequency regions. Moreover, we will design and implement an optical communication link to evaluate the BER performance under those conditions in future work.

In general, the wavy air–sea interface causes deterioration of the laser communication channel. The present study emphasizes the need to investigate techniques to mitigate that effect. Active adaptive optics should be considered to reduce the impact of wavefront distortion [56]. This approach can be extended, for example, by using micro-electromechanical system (MEMS) [57] deformable mirrors.

These characteristics of the oceanic facet’s behavior combined with laser propagation would facilitate future research to improve the performance of free space optical communication through the air–sea interface by tracking the optimal facet with the desired path to the receiver.

## Figures and Tables

**Table 1:** A summary of candidate models for oceanic waves.

Spectrum/Model	Description	References
Gerstner Waves (1802)	It is based on Navier–Stokes equation by describing a particle’s motion on the surface as a circular motion to provide an approximate to simulate the air–water interface.	[12,25]
Phillips (1954)	A fully developed sea is considered deep water. It is widely used in real-time simulation of oceanic waves.	[9,25,26]
Neumann (1955)	It is valid for only fully developed sea, and it is valid for only gravity waves regime.	[8]
Pierson–Moskowitz (1964)	It is based on the Phillips equilibrium range representing a fully developed sea. It is designed to describe gravity waves over infinite fetch.	[13,24]
JONSWAP (1964)	A modified Pierson–Moskowitz spectrum with enhanced peak and fetch dependent factors. It is valid only for limited fetch and infinite water depth.	[13,27,28]
TMA (1985)	Developed as an extension of the JONSWAP spectrum for finite water depth.	[26,29]
Majumdar & Brown (1992)	Probabilistic method applied to investigate the influence of the wavy air–sea interface on the laser beam transmission based on the Gram–Charlier model.	[30]
Apel (1994)	Modified version of the JONSWAP spectrum includes improved capillary and gravity–capillary wave predictions. It is developed for shallow water with short fetch winds (100–1000) m.	[10,31,32]
Elfouhaily (1997)	Using data observations from previous models, a unified directional spectrum for long and short wind-driven waves based on the Apel wave spectrum.	[33]

**Table 2:** The standard deviation for the refraction and slope angles in the along- and cross-wind directions with *d* = 0.3 cm.

Laser Beam Incident Angle (^o^) with *d* = 0.3 cm								
Standard Deviation of Slope Angle (^o^)					Standard Deviation of Refraction Angle (^o^)			
Wind Speed (m/s)	Along-Wind Direction		Cross-Wind Direction		Along-Wind Direction		Cross-Wind Direction	
	(0°)	(32°)	(0°)	(32°)	(0°)	(0°)	(32°)	(0°)
0.9	*0.04*	*0.07*	*0.03*	*0.03*	*0.01*	*0.04*	*0.01*	*0.02*
1.9	*0.21*	*0.17*	*0.2*	*0.09*	*0.07*	*0.10*	*0.06*	*0.05*
*3.3*	*4.18*	*2.13*	*1.66*	*0.78*	*1.39*	*1.27*	*0.55*	*0.47*

**Table 3:** The standard deviation for refraction and slope angles in along- and cross-wind directions with *d* = 0.8 cm.

Laser Beam Incident Angle (o) with *d* = 0.8 cm								
Standard Deviation of Slope Angle (^o^)					Standard Deviation of Refraction Angle (^o^)			
Wind Speed (m/s)	Along-Wind Direction		Cross-Wind Direction		Along-Wind Direction		Cross-Wind Direction	
	(0°)	(32°)	(0°)	(32°)	(0°)	(32°)	(0°)	(32°)
0.9	*0.04*	*0.05*	*0.04*	*0.03*	*0.01*	*0.03*	*0.01*	*0.02*
1.9	*0.2*	*0.10*	*0.17*	*0.07*	*0.07*	*0.06*	*0.06*	*0.04*
*3.3*	*1.73*	*1.05*	*0.83*	*0.38*	*0.57*	*0.63*	*0.27*	*0.23*

## Data Availability

Not applicable.
